# Greater improvement in *LRRK2* G2019S patients undergoing Subthalamic Nucleus Deep Brain Stimulation compared to non-mutation carriers

**DOI:** 10.1186/s12868-016-0240-4

**Published:** 2016-02-01

**Authors:** Massiva Sayad, Mohamed Zouambia, Malika Chaouch, Farida Ferrat, Mustapha Nebbal, Mohamed Bendini, Suzanne Lesage, Alexis Brice, Mohamed Brahim Errahmani, Boualem Asselah

**Affiliations:** Laboratory of Behavioral and Cognitive Neuroscience, FSB, University of Science and Technology Houari Boumediene, El Alia, Bab Ezzouar, BP 32, 16111 Algiers, Algeria; Department of BPO, Faculty of SNV, Blida 1 University, BP 270, 09000 Blida, Algeria; Department of Neurology, CHU Ben Aknoun, 16000 Algiers, Algeria; Department of Neurosurgery, Mohamed-Seghir Nekkache Military Hospital, Djasr Kasentina, Algiers, Algeria; Department of Neurology, Mohamed-Seghir Nekkache Military Hospital, Djasr Kasentina, Algiers, Algeria; UMR S 1127 PaInserm U 1127, Sorbonne University, UPMC Univ Paris 06, Paris, France; CNRS UMR 7225, Paris, France; The Brain and Spinal Cord Institute, 75013 Paris, France; Department of Chemistry, Blida 1 University, BP 270, 09000 Blida, Algeria

**Keywords:** Subthalamic Nucleus Deep Brain Stimulation (STN-DBS), Parkinson’s disease (PD), *LRRK2* (Leucine-rich repeat kinase 2) G2019S gene mutations, Mutation carrier (MC), Non-carriers (NC), Multiple correspondence analysis (MCA), Mini Mental State Examination (MMSE), Unified parkinson’s disease rating scale III (UPDRS-III), Schwab and England’s activities of daily living scale (S and E scale), Hoehn and Yahr scale (H and Y scale)

## Abstract

**Background:**

Bilateral subthalamic nucleus deep brain stimulation (STN-DBS) of parkinson’s disease (PD) patients has demonstrated to improve motor performance and to reduce dopa-induced dyskinesia. An association between the occurrence of dyskinesias and *LRRK2* (leucine-rich repeat kinase 2) G2019S gene mutations has recently been suggested. The aim of this study is to discover the impact of the G2019S mutation (with high incidence in the authors’ native Algeria) on the symptom response of PD in patients who underwent STN-DBS.

**Methods:**

We carried out a comparative statistical study for the clinical evaluation and neuropsychological assessment of 27 Algerian PD STN-DBS patients, both G2019S mutation carriers (MC) and non-carriers (NC). A multiple correspondence analysis (MCA) was then conducted to compare the results with those from groups of individuals with similar modalities.

**Results:**

The MCA revealed that MC and NC PD patients showed two different patterns of clinical evaluations. The group of idiopathic patients showed some differences compared to the clinical evaluations, depending on gender. No association was found between the G2019S mutation and the Mini Mental State Examination scores (MMSE), and MC patients appeared more susceptible to dyskinesia than NC patients. In NC patients, we found two cases with Parkin mutations who had a different “honeymoon” period and different initial symptoms. The results showed considerable improvement of motor unified parkinson’s disease rating scale III (UPDRS-III) in a situation of stimulation without medication in the MC patients with a percentage of improvement (51.1 %) over the required 30 % compared to the NC patients (25.5 %). The same result was observed for the Schwab and England’s activities of daily living scale (S and E scale), which thus demonstrated a greater effectiveness of DBS for MC patients than for NC patients. However, the Hoehn and Yahr scale (H and Y Scale) showed the same significance in a situation of stimulation for MC and NC patients. In this later group, the best scores of UPDRS-III were observed for patients with the Parkin mutation before they underwent surgery.

**Conclusions:**

This study shows that surgical treatment probably has a more significant impact on *LRRK2* G2019S MC than on idiopathic patients.

## Background

Parkinson’s disease (PD) is considered to be a multifactorial etiology disease, and, in most cases, the result of multiple factor effects, either genetic or environmental. Genetic susceptibility factors have also been implicated in idiopathic forms of PD. Two independent studies [1, 2] have identified a mutation in the *LRRK2* gene that encodes a protein called dardarin. So far, seven validated missense mutations have been reported in European and North American PD populations. A common G2019S mutation was found to account for approximately 5 % of familial cases, but for markedly more cases in North African (≈40 %) and Jewish populations [3, 4]. In a large Algerian PD cohort, a comparative study of clinical aspects and progressive parkinsonian signs between G2019S MC and NC patients showed that this mutation is probably associated with the occurrence of dyskinesias, suggesting a genetic predisposition for these complications [5].

Although these dyskinesias are difficult to treat, DBS has shown to be effective, probably by the removal of abnormal action potential profiles [6]. Currently, DBS of the STN is regarded as the best surgical treatment for L-dopa-responsive symptoms of PD at the stage of motor fluctuations [7, 8]. However, with the progression of the disease, and despite initial improvement, the response of the axial symptoms to L-dopa and therefore to the STN stimulation deteriorates progressively [9, 10].

The DBS indication follows CAPSIT-PD recommendations (Core Assessment Program for Surgical Interventionnal Therapies in PD) [11], which additionally recommends a disease duration of a minimum of 5 years and a good sensitivity to L-dopa. There does not appear to be a genetic test to determine the criteria for inclusion or exclusion of patients suffering from PD and DBS, whereas the share of the G2019S mutation, given its frequent occurrence in our country, in the earliest occurrence of dyskinesias and the progression of the disease may be essential to be able to predict the long-term effectiveness of DBS treatment. To do this, we have opted for a MCA, which is a multivariate analysis that allowed us to graphically analyze and describe remarkable combinations between the genetic and clinical parameters of different PD patients having undergone DBS.

## Methods

This study focused on 27 PD patients who underwent bilateral STN-DBS and parameter conditions were tested under simple blind randomized conditions.

Each PD patients was implanted at the neurosurgical department in which he was followed (Salim Zemirli Hospital of Algiers, the Mohamed-Seghir Nekkache Military Hospital of Algiers and Frantz Fanon Hospital of Blida) from April 2006 to October 2012.

The follow-up of the patients was relayed to specialized neurosurgeons, neurologists and psychologists in their relevant fields of expertise at their respective hospitals and at the Neurology department of Ben Aknoun Hospital. The raters did not know the patients’ genetic status either before or after surgery.

All PD patients accepted and gave informed consent to take part in this anonymous study. For each patient, we completed a questionnaire with demographic data such as gender, date of birth and profession.

### STN-DBS surgical procedure

The CAPSIT-PD recommendations for STN-DBS were followed in all patients for the three neurosurgical departments. The most important criteria for STN-DBS eligibility are: patients with correct diagnosis of idiopathic PD with age at surgery <60 years, good sensitivity to L-dopa with presence of dyskinesias.

The same surgical technique and equipment for the bilateral implantation was used for all patients. Coordinates of the targets were defined by imaging methods such as computerized tomography scans (CT-scan) and magnetic resonance imaging (MRI). The location of the leads was checked at the end of surgery by bidirectional skull X-ray in the stereotactic frame. Each contact of the lead was tested in monopolar mode at a predefined pulse width (typically 60 μs for most DBS devices) and frequency (typically 130 Hz) and the amplitude was increased carefully until the first stimulation-induced adverse effect appeared. Conversely, if the electrode had not been well placed, the neurologist was able to test different combinations, including bipolar settings. The stabilization period was dedicated to the gradual adjustment of stimulation parameters and medication. It tended to be completed within 3–6 months after surgery.

In this study, we report surgery-related complications occurring after STN-DBS.

### Clinical study

We prepared a form that was filled in by the neurologist and that recorded the clinical forms of the disease, age and clinical signs from the onset, as well as treatment, the honeymoon period, progression of the disease, DBS duration, Unified Parkinson’s Disease Rating Scale III (UPDRS-III), Hoehn and Yahr stage (H and Y stage), Schwab and England quality of life scale (S and E scale) in four medication situations and DBS as medication-stimulation Off–Off (before surgery, at least 12 h after the last dose of L-dopa), On–Off (before surgery, at 1 h after the administration of L-dopa), Off–On (at least 12 h after the last dose of L-dopa and 1 h after the stimulator was turned on) and On–On (24 months postoperatively, at 1 h after the administration of L-dopa and 1 h after the stimulator was turned on) in the same time intervals for all patients. The UPDRS-III is more suitable for motor evaluation. It is used to quantify therapeutic improvement and progression of the disease. The H and Y stage allows having a global vision of the disease while S and E scales enable the rating of the degree of autonomy of PD patients [12].

The so-called “honeymoon period” corresponds to the period of improved survival, when PD patients are most responsive to L-dopa and ends when treatment-associated complications such as dyskinesias start.

### Neuropsychological assessment

The Mini Mental State Examination (MMSE) evaluates memory and orientation in time and space, attention and basic arithmetic functions, memory retention, language and constructive praxis.

### Screening of the *LRRK2* G2019S, Parkin, Pink1 and DJ-1 mutations

Blood samples were collected and family trees were made for each patient. We took care to mention the family history of the disease, the number of deceased relatives who had suffered from PD (Lohman) and the presence or absence of consanguinity.

DNA was extracted with the phenol/chloroform technique and then stored at −20 °C until use.

The G2019S mutation in exon 41 of the *LRRK2* gene (rs34637584) results in a change from a guanine (GGC) to an adenine base (AGC) at codon which changes a glycine to a serine at position 2019.

Also, we analyzed the recessive genes (Parkin, Pink1 and DJ-1) for NC patients with linkage study. This study was performed by analyzing of microsatellite located in or near gene then by sequencing of individuals having all of the microsatellite markers homozygous. The thermocycler used was BIO-RAD (DNA Engine, Peltier Therma Cycler). We screened all 12 exons for Parkin mutations, 8 exons for PINK1 mutations (Exon 1 was tested in two overlapping parts 1a and 1b) and 7 exons for DJ1 mutations.

The genotyping was performed in the Brain and Spinal Cord Institute of Paris (France) for all patients. We used the TaqMan assay (Applied Biosystems™ kit) on the Applied Biosystem 3739 DNA Analyzer sequencer (HITACHI). Any changes were checked by sequencing using the SeqScape v2.6 software.

### Statistical analysis

The Friedman’s two-way analysis of variance (ANOVA) by ranks was performed as test of comparisons in paired sample populations subjected to four different situations of medication and stimulation.

MCA is a multivariate analysis based on measurements of several variables with two or more modalities. The expected links were highlighted by analyzing and graphically descriptions, on a graph with the two maximal variance factors, the groups of individuals with similar modalities. The interpretation of MCA is generally based on parameters showing the best qualities of representation (cos^2^ factor). Means are shown as $\overline{\text{x}} \pm {\text{SD}}$ (SD is the standard deviation).

## Results

The 27 Parkinsonian patients (17 males and 10 females (sex ratio 1.70)), who underwent DBS at STN level, had an average age of 55.4 ± 8.0 years and an average age at the onset of the disease of 40.2 ± 8.7 years. There were no significant differences between males and females with respect to age at time of examination and age at onset (Table 1).

**Table 1:** Clinical characteristics of 27 STN-DBS patients in relation to gender

	Means	Min	Max	Men (n = 17)	Women (n = 10)	p
Age groups (years)	55.4 ± 8.0	41	69	56.4 ± 9.5	53.8 ± 4.3	0.34
Age at onset of PD (years)	40.2 ± 8.7	25	55	40.9 ± 10.7	39.0 ± 3.9	0.34
Duration of disease (years)	15.3 ± 3.0	10	20	15.5 ± 3.3	14.9 ± 2.5	0.60
Duration of honeymoon (years)	4.5 ± 2.3	0.25	10	4.5 ± 1.8	4.6 ± 3.0	0.91
Duration of STN-stimulation (years)	4.7 ± 1.4	2.6	8	4.9 ± 1.5	4.2 ± 1.2	0.18

*PD* parkinson’s disease, *STN* subthalamic nucleus, *Min* minimum, *Max* maximum

### Age groups and gender

**Fig. 1:**
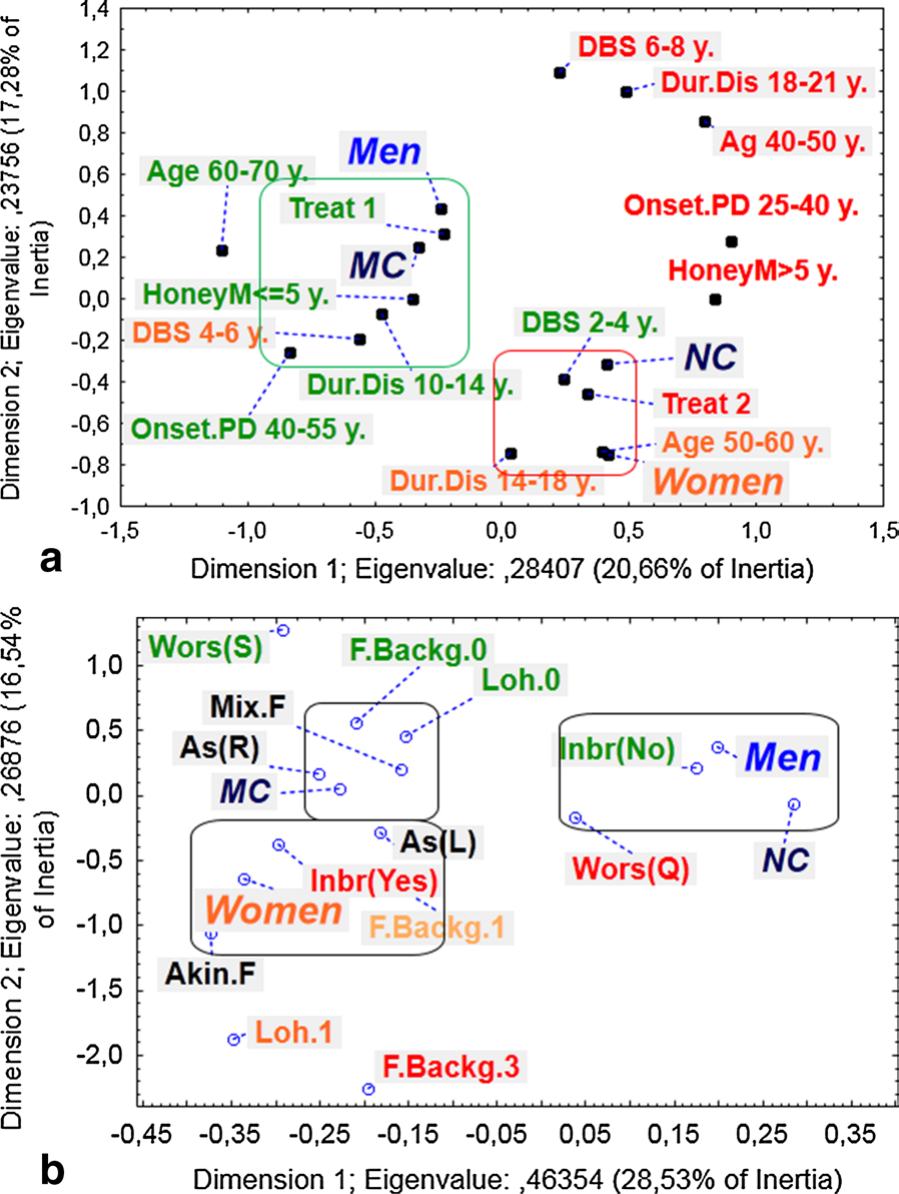
Multiple correspondence analysis (MCA) between clinical and genetic variables. **a** On the *left* side: *MC* mutation carriers, *NC* non-carriers, Age: age classes (years), Onset.PD: Age at onset of PD (years), *Dur.Dis* duration of disease (years), *Treat* Treatment (Treat 1 <=500 mg/day, Treat 2 > 500 mg/day), *DBS* duration of deep brain Stimulation (years), *HoneyM* duration of honeymoon (years). **b** On the *right* side: *MC* mutation carriers, *NC* non-carriers, *F.Backg* family background of the disease, *Wors* Worsening (Q quick, S slow), *Inbr* inbreeding, asymmetry: As(R) right asymmetry, As(L) left asymmetry, disease form: *Mix.F* mixed form, *Akin.F* akinetic rigid form, Lohman: Loh0, Loh1, Loh2, several deaths. The *green*, *orange* and *red colors* were respectively for high, intermediate and high modalities of the variables

The link between the age groups and gender was slightly significant (χ^2^ = 6.15, p = 0.046), with men predominating in the highest age groups (60–70 years), and women in the intermediate age groups (50–60 years). This was highlighted and confirmed by the MCA (Fig. 1a).

### Age at examination and age at onset of PD

There was no connection between age at onset and presence of the G2019S mutation (χ^2^ = 0.90, p = 0.34). However, there was a tendency towards a link between so-called honeymoon period (up to 5 years and more than 5 years) and the presence of the mutation (χ^2^ = 4.30, p = 0.04 < 0.05), there were significantly more G2019S MC patients with shorter honeymoon period than the NC patients. The results also showed that 70.4 % of the patients had a honeymoon period shorter than 5 years when taking L-dopa.

### Psychological assessment

The χ^2^ test was not significant (p = 0.60 ≫ 0.05), so there was no significant difference of MMSE score between the G2019S MC and NC patients (25.3 ± 3.1 and 26.2 ± 2.7 respectively) (Table 2).

**Table 2:** Clinical comparative study of 27 STN-DBS patients in relation with presence/absence of G2019S mutations

	MC (n = 15)	NC (n = 12)	p
Age groups (years)	56.2 ± 8.8	54.5 ± 7.1	0.59
Age at onset of PD (years)	40.1 ± 9.4	40.3 ± 8.2	0.97
Duration of disease (years)	16.1 ± 3.0	14.3 ± 2.7	0.10
MMSE (maximum score 30)	25.3 ± 3.1	26.2 ± 2.7	0.60
Duration of honeymoon (years)	3.8 ± 1.7	5.4 ± 2.6	0.08
Duration of STN-stimulation (years)	5.1 ± 1.4	4.1 ± 1.3	0.09

*PD* parkinson’s disease, *MMSE* Mini Mental State Examination, *STN* subthalamic nucleus, *MC* mutation carriers, *NC* non mutation carriers

In our series, 74.1 % had a normal MMSE (25–30). In the remaining 25.9 %, there were five female and two male patients with a low sociocultural level and one patient with aphasia.

### Lead location and DBS complications

Of the 54 contacts used for bilateral STN-DBS (for 27 patients), 53 contacts were localized in or near the STN. We registered one contact in the right side of brain to be localized outside of the STN. In three cases, we encountered DBS complications (one MC patient and two NM patients) with moderate pneumocephalus in the frontal region and three other cases (one MC patient and two NM patients) had stimulator infections.

Comparative evaluations of the optimal stimulation parameters are: pulse width 60 μs, frequency 130 Hz, amplitude means are 2.32 ± 0.50 V at left and 2.35 ± 0.54 V at right side STN.

### Genetic study

The genetic study revealed the presence of the G2019S mutation in 55.6 % of PD patients who had undergone DBS stimulation with an average age at examination of 56.2 ± 8.8 years and an average age at the onset of the disease of 40.1 ± 9.4 years. The NC patients had the same average age at examination and average age at onset (54.5 ± 7.1 and 40.3 ± 8.2 years respectively). The average duration of the honeymoon period (period during which there is a sustained response to dopaminergic treatment and the patient does not have a dyskinesia) was slightly shorter in patients with the MC (3.8 ± 1.7 years) than those with NC (5.4 ± 2.6 years) (Table 2).

Among all the NC patients, we found only two cases (16.66 %) of Parkin mutation (heterozygous c. 1204C>T in exon 11 and c. 458C>G in exon 4) with an age at onset of PD of 48 years, different initial symptoms and duration honeymoon (Table 3).

**Table 3:** Case reports of patients with Parkin mutation

	Case 1	Case 2
Gene and	Parkin	Parkin
Exon with mutation	Exon 4	Exon 11
Mutation type	Heterozygous	Heterozygous
	c. 458C>G	c. 1204C>T
Protein change	P153R	p. Arg402Cys
Gender	Male	Male
Age at onset	48 years	48 years
Disease duration	10 years	13 years
Family history of Parkinsonism	Negative	Negative
UPDRS-III Off–Off scores	46/108	49/108
UPDR-III On–Off scores	28/108	32/108
UPDRS-III Off–On scores	51/108	51/108
UPDRS-III On–On scores	30/108	47/108
Initial symptoms	Resting tremor in left hand, dyskinesias 4 years after initiation of treatment.	Muscle stiffness in both upper limbs, dyskinesias 7 years after initiation of treatment.

### Clinical UPDRS-III, S and E scales and H and Y stages

The UPDRS-III of MC patients in Off–Off medication-stimulation situations had an average score of 55.8 ± 16.4, which was significantly higher than the scores in three other cases (average score <28; Table 4). Indeed, the mean values of UPDR-III decreased from 55.8 ± 16.4 (in Off–Off situation) to 27.3 ± 20.6 (in Off–On medication-stimulation), therefore, this is an UPDRS-III improvement of 51.1 % compared to the value Off–Off (very highly significant p = 0.000046 ≪ 0.001).

**Table 4:** Clinical UPDRS-III, S and E scales and H and Y stages scores of PD MC and NC patients undergoing STN-DBS (mean ± SD)

Medication stimulation	UPDRS-III		S and E scale		H and Y scale	
situations	MC	NC	MC	NC	MC	NC
Off–Off	55.8 ± 16.4	51.7 ± 14.4	0.43 ± 0.18	0.52 ± 0.14	3.50 ± 0.86	3.54 ± 0.65
On–Off	25.0 ± 13.2	30.6 ± 16.7	0.70 ± 0.13	0.69 ± 0.16	2.36 ± 0.66	2.87 ± 0.90
Off–On	27.3 ± 20.6	38.5 ± 16.6	0.60 ± 0.21	0.60 ± 0.26	2.36 ± 0.69	2.16 ± 0.91
On–On	19.7 ± 18.8	18.8 ± 12.5	0.85 ± 0.16	0.89 ± 0.10	2.13 ± 0.71	1.91 ± .99
p	0.00005	0.0003	0.00001	0.00106	0.00002	0.00091

Unified parkinson’s disease rating scale III (UPDRS-III); Hoehn and Yahr scale (H and Y scale); Schwab and England’s activities of daily living scale (S and E scale)

*MC* mutation carriers, *NC* non mutation carriers

For NC patients, the test was very highly significant too (p < 0.001). The UPDRS-III in Off–Off medication-stimulation had an average score of 51.7 ± 14.4 again, and was significantly higher than the other three situations (average score <39) (Table 4). The mean values of UPDR-III decreased by 51.7 ± 14.4 (in Off–Off situations) to 38.5 ± 16.6 (in Off–On medication-stimulation situations). So, this is an UPDRS-III improvement of 25.5 % (non significant p = 0.09). The best scores of UPDRS-III of Parkin mutation patients were observed in a situation of medication (On–Off situation) compared with a postoperative situation (Table 3).

The comparison of the four UPDRS-III situations showed more significance in MC patients (Fridman’s ANOVA p = 0.00005 ≪ 0.001) than in NC patients (p = 0.0003) (Table 4).

Similar results were found for the four S and E scale situations, which showed more significant variations in MC patients (p = 0.00001 ≪ 0.001) compared to NC patients (p = 0.001) (Table 4). The S and E scale for MC patients in Off–On medication-stimulation had an average score of 0.6 ± 0.21, while the Off–Off situation had the lowest at 0.43 ± 0.18 (slightly significant p = 0.036). Similar results were found for the NC patients, with an average score of 0.6 ± 0.26 for the Off–On medication-stimulation, while the lowest was at 0.53 ± 0.14 for the Off–Off (non significant p = 0.41).

The comparison of the four H and Y stage situations showed more significance variations in MC patients (p = 0.00002 ≪ 0.001) compared to NC patients (p = 0.0009) (Table 4). The H and Y scale in the Off–Off situation had an average score of 3.50 ± 0.87, which was significantly higher than in the three other cases (average score <2.40). The H and Y stage for MC patients in Off–On medication-stimulation had an average score of 2.36 ± 0.69, while the Off–Off situation had the highest at 3.50 ± 0.87 (very highly significant p = 0.0003 ≪ 0.001).

For NC patients, the test was very highly significant too (p = 0.0009 < 0.001). The H and Y scale in the Off–Off situation had an average score of 3.54 ± 0.66, which was significantly higher than in the three other cases (average score <2.90). The H and Y stage for NC patients in Off–On medication-stimulation had an average score of 2.16 ± 0.91, while the Off–Off situation had the highest at 3.54 ± 0.66 (very highly significant p = 0.00005 ≪ 0.001).

### Multiple correspondence analysis (MCA)

Figure 1a shows lower modalities of the variables on the left side for men and higher modalities of the variables on the right side for women.

The MCA provided added value and shows that NC women, who were in the average age groups (50–60 years old), had a disease duration of between 14 and 18 years, treated with high doses (>500 mg/day).

Conversely, MC men and L-dopa dose was reduced to below 500 mg/day after DBS, were in the 60–70 years age group and had developed the disease later than women (40–55 years old).

The distinction between the two groups (enclosed at left side of Fig. 1b) was fairly clear and shows that MC patients have no family history of the disease and tended to have a mixed form of the disease with right asymmetry predominance. However, the Clustering in the left lower figure is characterized by the presence, in female patients, of an akinetic-rigid form of the disease, and a family history of the disease with consanguinity.

The third group (on the right side of Fig. 1b) was that of the non-G2019S mutation carrying male patients, with no consanguinity and rapid worsening of the disease.

## Discussion

The average age at onset of the disease was 40.2 ± 8.7 with 48.1 % in the patients having early onset of the disease (25–40 years old) and 51.9 % with a disease onset age of 40–70 years old. This is in contrast with the literature, where PD is rarely observed to occur before 40 years of age (early PD onset represents less than 10 % of the cases) and 80 % of the cases start between the ages of 40 and 75 years [13]. This may be explained by the prevalence of a common idiopathic form in our series.

There were more males than females in our series, with a gender ratio of about 1.7. This difference would be justified, according to Moisan and Elbaz [14], who reported a gender ratio of 1.5 to the more frequent occupational exposure of men to neuro-toxic substances, and the neuro-protective effect of estrogen or a genetic factor linked to the X chromosome on women. Indeed, 70 % of the women in our series did not have a profession, whereas the men all did, be it manual laborers or white collar workers. In our series, female gender was more common amongst MC than NC (60 vs. 40 %). Indeed, it has been shown that PD patients with *LRRK2* mutations are more likely to be women, suggesting a high genetic load versus idiopathic [15]. In addition, we should not exclude the fact that these patients were pre-selected for DBS after the processing of their files and that the gender criterion did not appear in the selection criteria.

We experienced a few complications with the DBS procedures. These complications are explained by the duration of surgery and the numbers of electrode penetration [16, 17]. The poor outcome of STN-DBS in PD is generally related either to incorrect implantation or to hardware failure [18]. Incorrect lead placement, which may be due to a number of factors, including stereotactic inaccuracy, poor initial targeting or loss of cerebrospinal fluid during surgery, may have led to brain shift [19].

Monopolar stimulation (amplitude 2.32 ± 0.50 V at left and 2.35 ± 0.54 V at right side STN, width 60 μs, frequency 130 Hz) has been used in most patients by most studies [20, 21]. Therapeutic amplitudes for DBS normally range between 1 and 4 V and the pulse width for stimulation of STN is 60 μs. Also, the frequency is 130 Hz in order to reach maximal benefit with minimal battery drain [22].

The presence of the G2019S mutation was observed in 15 patients (i.e. 55.6 % of the cases), including 8 sporadic and 7 familial PD patients, all heterozygous. This number is higher than the one reported by Belarbi and collaborators [5] for the Algerian population; with their 34 MC patients (i.e. 32.4 %) of which 28 were sporadic and 6 were familial PD patients. It was heterozygous in the familial form and sporadic in almost all cases.

The majority of MC patients had late onset of the disease (after 40 years old) and a relatively short honeymoon period (5 years), while NC patients were mostly found to have early onset (between 20 and 40 years old) and a longer honeymoon period (between 6 and 10 years). According to Belarbi and collaborators [5], the comparison of the clinical and evolutionary signs in G2019S MC and NC in PD showed a similarity in the clinical signs but the motor complications of the treatment induced by L-dopa were more frequent for the MC patients. Thus, this mutation appeared to be associated with the occurrence of dyskinesias given the high frequency of its complications in MC, suggesting a genetic predisposition to these complications.

Two NC patients (16.66 %) showed Parkin mutations, one was an early-onset case (48 years). Indeed, we reported in the literature that the Parkin mutation has been identified in several families with autosomal recessive early-onset Parkinsonism [23, 24] and frequency has been estimated at 10–25 % [25]. In contrast, the clinical characteristics of some European and North African patients with Parkin mutations were characterized with an age at onset of up to 58 years [24, 26]. The “honeymoon period” and initial symptoms were different in two patients with the Parkin mutations (the heterozygous c.1204C>T mutation in exon 11 and c.458C>G mutation in exon 4 of Parkin). In the literatures, the roles of Parkin heterozygous mutations at risk for PD have not been conclusively shown [27, 28]. Nevertheless, in a recent study, a disease-associated heterozygous mutation of Parkin was found in one patient with early-onset, slowly progressive Parkinson’s disease with Lewy bodies and very late development of dementia [29]. However, the number of patients with Parkin mutation in our series so far is small and the correlations between genotype and phenotype are uncertain.

The MCA revealed the presence of some clinical settings common to patients with the *LRRK2* G2019S mutation compared to NC. The G2019S patients were probably characterized by the mixed form of the disease with predominance of right side asymmetry.

On the other hand, NC patients may show differences with respect to certain clinical gender parameters. Males with no family consanguinity and in an age group between 60 and 70 years possibly developed the disease later than females (40–55 years old). At the same time, females with family consanguinity and in age group between 50 and 60 years old likely developed the disease earlier (20–40 years old), with an akineto-rigid form with predominance of left side asymmetry.

The literature shows that there is no associated phenotype related to MC. Although the clinical evaluation resembles that of a typical Parkinsonian syndrome, the age of onset of the disease is remarkably variable, ranging from 35 to 78 years [4]. Moreover, recent study reveals that G2019S MC patients is similar to NC PD patients but is characterized with more frequent lower extremity involvement at onset and postural instability and gait difficulty without the associated cognitive impairment [30].

It is also known that during the pathological neurodegeneration process, lesion distribution, initially unbalanced, occurs at the same time as the occurrence and worsening of clinical symptoms. Thus, this evolution of the pathological process can sometimes lead to the dominance of a different subtype of clinical expression of the initial diagnosed subtype [31, 32]. A recent neuropathological study also showed a pleomorphic phenotype in this MC [33]. This clinical and pathological variability suggests that the G2019S mutation plays a role in several neurodegeneration interactions.

No significant difference was observed in MMSE between MC patients and NC. In fact, Goldwurm and collaborators [34] reported that there were no significant cognitive dysfunctions in MC and NC. Lesage and collaborators [4] also reported an insignificant difference in MMSE in a French and North African population. On the other hand, Barth and collaborators [5] reported the lowest MMSE in MC patients, which the authors attributed to the small size of the sample in their study.

The UPDRS-III graphics show a marked improvement in the clinical spectrum of the PD patients. The percentage of improvement of 51.1 % for the G2019S MC patients in Off–On medication-stimulation situations was above 30 %. It is empirically considered that an improvement of more than 30 % of the UPDRS-III is clinically significant [12]. In NC patients, the percentage of improvement under stimulation without drug therapy was below the required 30 % (25.5 %). The S and E scales as well as the H and Y stages in various situations of medication and stimulation also showed a marked improvement during Off–On medication-stimulation. This result demonstrates the effectiveness of DBS. It is known that DBS at the STN level largely improves the quality of life of patients because it acts on all aspects of the PD triad: tremor, rigidity and akinesia [35]. According to a study dating from 2011, the improvement in UPDRS-III was 41 % compared with the control group 12 months after surgery [36]. The first beneficial effects occur in the minutes following the start of the stimulation [35]. In the long run, DBS is more stable than L-dopa treatment, and motor fluctuations and dyskinesia become minor [37].

However, the differences in the UPDRS-III, the S and E scales as well as the H and Y stages in the four situations for MC patients were more significant than for NC patients. Also, the comparison between MC and NC in a situation of stimulation and a situation without medication-stimulation in the UPDRS-III and the S and E scales showed the best response for the MC patients. This means that the G2019S MC patients have a better response than NC patients. In fact, it is known from the literature that patients with *LRRK2* mutations are good candidates for STN stimulation [38, 39], but limited series are available so far. In contrast, other studies suggested that no influence by the *LRRK2* G2019S mutation exists on STN-DBS results [38, 40, 41]. However, the series reported in the literature are lower than our series (15 MC patients). It is supposed that this mutation makes PD patients more vulnerable to dyskinesias [5] or to some deleterious reorganization of corticostriatal efferents that would be modulated by the STN-DBS [39]. This leads us consider that this mutation may have impact on the progression and response to STN-DBS. Further larger studies are needed to confirm these findings.

The patients with the Parkin mutation have the best UPDRS-III scores in the On–Off medication-stimulation situation compared to other situations. According to the literatures, the effect of DBS did not differ between patients with and without Parkin mutations [42, 43].

## Conclusion

In our study, MCA revealed the presence of two distinct groups: MC and NC had two different clinical evaluations. The MC patients were probably characterized by the mixed form of the disease, with a predominance of right side asymmetry. In the NC group, men from non-consanguineous families and with an age (at examination) ranging between 60 and 70 years may develop the disease later than the women (40–55 years).

Other noteworthy findings were a shorter honeymoon period of MC patients compared to the NC. Moreover, in the later group, we found two patients with Parkin mutations who had a different honeymoon period and different initial symptoms. Also, the results showed that the G2019S mutation was not associated with MMSE scores.

Other significant results were a clear improvement in the UPDRS-III more for MC than for NC patients who underwent stimulation with percentages of improvement over the required 30 % for MC patients only (51.1 and 25.5 % respectively). We found the same result for the S and E scales, which thus demonstrated the effectiveness of DBS for MC patients more than for NC patients. This indicates that, compared to NC patient, MC patients are probably the best candidates for STN-DBS. On the other hand, the best scores of UPDRS-III observed in situations where Parkin mutation patients received medication suggest that STN-DBS probably did not benefit these patients.

### Ethics approval and consent to participate

All participants provided written informed consent for the publication of individual clinical details.

The study was approved by the institutional review boards and ethical approval was obtained from Ethics board and Academic Deontology of the Ministry of Higher Education and Scientific Research.

## Abbreviations

Akin.F: akinetic rigid form
ANOVA: analysis of variance
As(L): left asymmetry
As(R): right asymmetry
CAPSIT-PD: Core Assessment Program for Surgical Interventionnal Therapies in PD
CT-scan: computerized tomography scans
Dur.Dis: duration of disease
F.Backg: family background of the disease
H and Y Scale: Hoehn and Yahr scale
HoneyM: duration of honeymoon
Inbr: inbreeding
L-dopa: levodopa
Loh: Lohman
*LRRK2*: leucine-rich repeat kinase 2
Max: maximum
MC: mutation carrier
MCA: multiple correspondence analysis
Min: minimum
Mix.F: mixed form
MMSE: Mini Mental State Examination
MRI: magnetic resonance imaging
NC: non-mutation carriers
Onset.PD: age at onset of PD
PD: parkinson’s disease
S and E scale: Schwab and England’s activities of daily living scale
STN-DBS: subthalamic nucleus deep brain stimulation
Treat: treatment
UPDRS-III: unified parkinson’s disease rating scale III
Wors.Q: worsening quick
Wors. S: worsening slow
